# Supplementary material for: Co‐recruitment analysis of the CBL and CBLB signalosomes in primary T cells identifies CD5 as a key regulator of TCR‐induced ubiquitylation
Source: Mol Syst Biol. 2016 Jul 29;12(7):876. doi: 10.15252/msb.20166837 (PMC4965873; doi:10.15252/msb.20166837)
Supplement: Supplementary file 1 — Expanded View Figures PDF [file MSB-12-876-s001.pdf]

Expanded View Figures

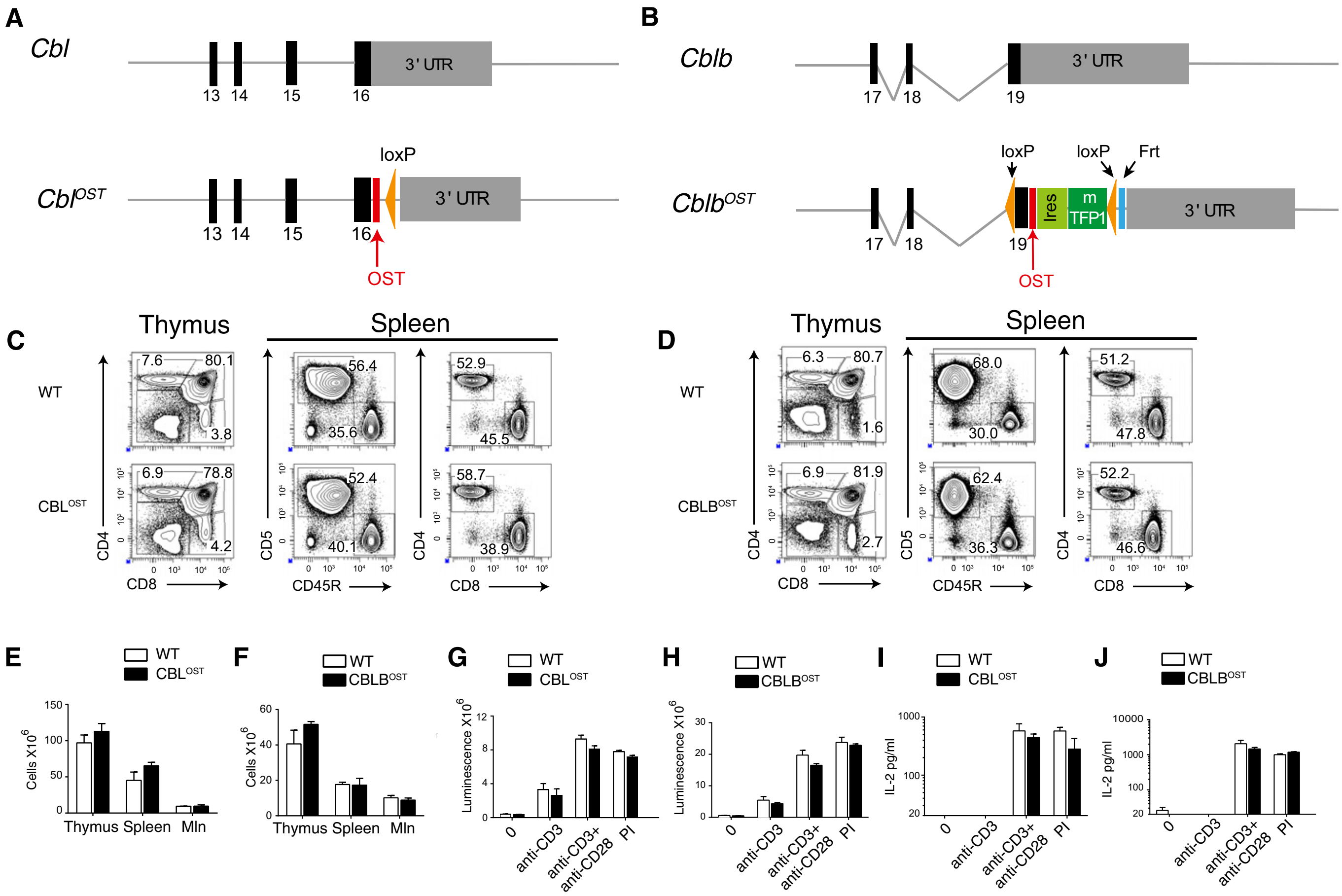

Figure EV1. T cells from CBL^{OST} and CBLB^{OST} mice develop and function normally.

- A Structure of the 3′ end of the wild-type *Cbl* allele and of the targeted *Cbl*^{OST} allele following homologous recombination and CRE-mediated excision of the loxP-neo^{r}-loxP cassette. Exons are shown as filled black boxes and numbered. In the *Cbl*^{OST} allele, the One-STrEP-tag (OST) is shown in red and the remaining loxP site in orange.
- B Structure of the 3′ end of the wild-type *Cblb* allele and of the targeted *Cblb*^{OST} allele following homologous recombination and FLP-mediated excision of the frt-neo^{r}-frt cassette. Exons are shown as filled black boxes and numbered. In the *Cblb*^{OST} allele, the One-STrEP-tag (OST) is shown in red, the IRES-mTFP1 cassette in green, the two loxP sites in orange, and the remaining frt site in blue.
- C Flow cytometry analysis of thymus and spleen from wild-type (WT) and CBL^{OST} mice for expression of CD4 versus CD8 and CD5 versus CD45R. Numbers adjacent to outlined areas indicate percentage of cells.
- D Flow cytometry analysis of thymus and spleen from wild-type (WT) and CBLB^{OST} mice for expression of CD4 versus CD8 and CD5 versus CD45R. Numbers adjacent to outlined areas indicate percentage of cells.
- E Cellularity of thymus, spleen, and pooled mesenteric lymph nodes (Mln) from wild-type (WT) and CBL^{OST} mice. Data are expressed as mean value ± SEM.
- F Cellularity of thymus, spleen, and pooled mesenteric lymph nodes (Mln) from wild-type (WT) and CBLB^{OST} mice. Data are expressed as mean value ± SEM.
- G ATP content of CD4^{+} T cells purified from WT and CBL^{OST} mice and activated for 48 h with PMA and ionomycin (PI) or with plate-bound anti-CD3 (0.3 μg/ml) in the presence or absence of soluble anti-CD28 (1 μg/ml). ATP content is directly proportional to the numbers of proliferating cells in the well and assessed by luminescence. Data are expressed as mean value ± SEM.
- H ATP content of CD4^{+} T cells purified from WT and CBLB^{OST} mice and activated for 48 h with PMA and ionomycin (PI) or with plate-bound anti-CD3 (0.3 μg/ml) in the presence or absence of soluble anti-CD28 (1 μg/ml), assessed by luminescence. Data are expressed as mean value ± SEM.
- I IL-2 in supernatants of WT and CBL^{OST} CD4^{+} T cells activated as in (G). Data are expressed as mean value ± SEM.
- J IL-2 in supernatants of WT and CBLB^{OST} CD4^{+} T cells activated as in (H). Data are expressed as mean value ± SEM.

Data information: Data in (C-J) are representative of at least three experiments with at least two mice per genotype.

 © 2016 The Authors

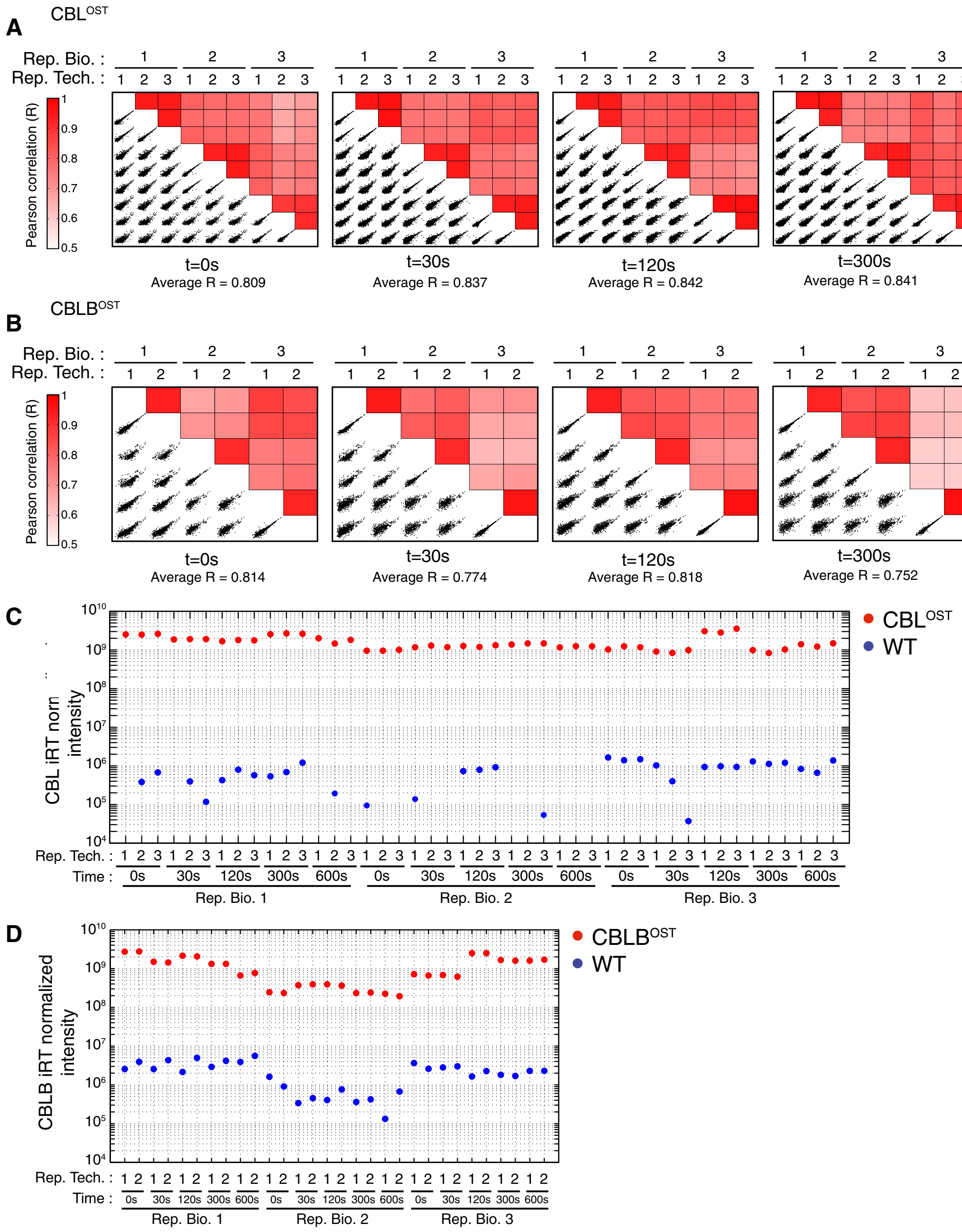

Figure EV2. Assessment of biological and technical variability across samples.

A, B For CBL-OST (A) and CBLB-OST (B) samples, the variability between samples corresponding to a given condition of activation was estimated by computing the Pearson correlation coefficient from log-transformed intensities—normalized using indexed Retention Time peptide intensities (iRT Kit; Biognosys)—of all detected proteins for all pairs of technical and biological replicates (denoted as Rep. Tech. and Rep. Bio., respectively). For each condition of activation (i.e. prior to $t = 0$ s or following TCR engagement for $t = 30$ s, $t = 120$ s, $t = 300$ s, and $t = 600$ s), scatter plots of log-transformed and iRT-normalized intensities for all pairs of technical and biological replicates are represented along with the corresponding Pearson correlation coefficients (R). The average Pearson correlation coefficient across all pairs of technical and biological replicates (denoted as Average R) is also indicated for each time point. Strong correlations exist between technical replicates corresponding to the same biological replicate, thereby illustrating the reproducibility of the LC-MS measurement.

C, D The iRT-normalized intensity of the CBL (C) or CBLB (D) bait proteins is represented for all the LC-MS runs corresponding to CBL-OST, CBLB-OST, and wild-type (WT) backgrounds. In each sample, a strong enrichment of the bait protein is observed in AP-MS purifications performed in the CBL-OST or CBLB-OST backgrounds as compared to control purifications performed in the WT background. In the case of control purifications from WT backgrounds, missing values are not represented.

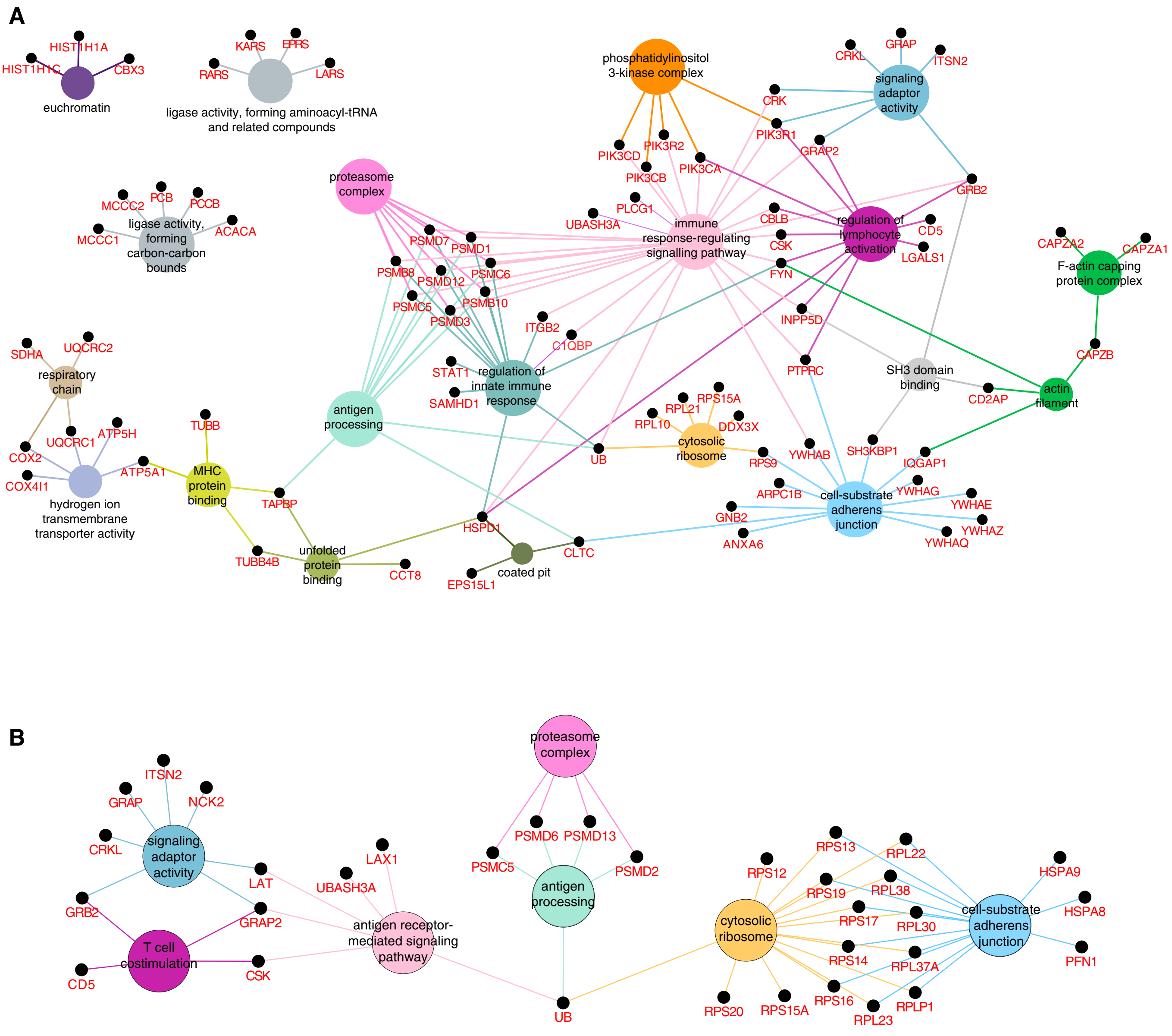

Figure EV3. GO term enrichment analysis of CBL and CBLB signalosomes.

A, B Organization of the CBL (A) and CBLB (B) signalosomes according to GO annotations. Enriched GO terms were extracted from the list of interacting partners identified in this study using the ClueGO plugin (http://apps.cytoscape.org/apps/cluego) on Cytoscape.

© 2016 The Authors

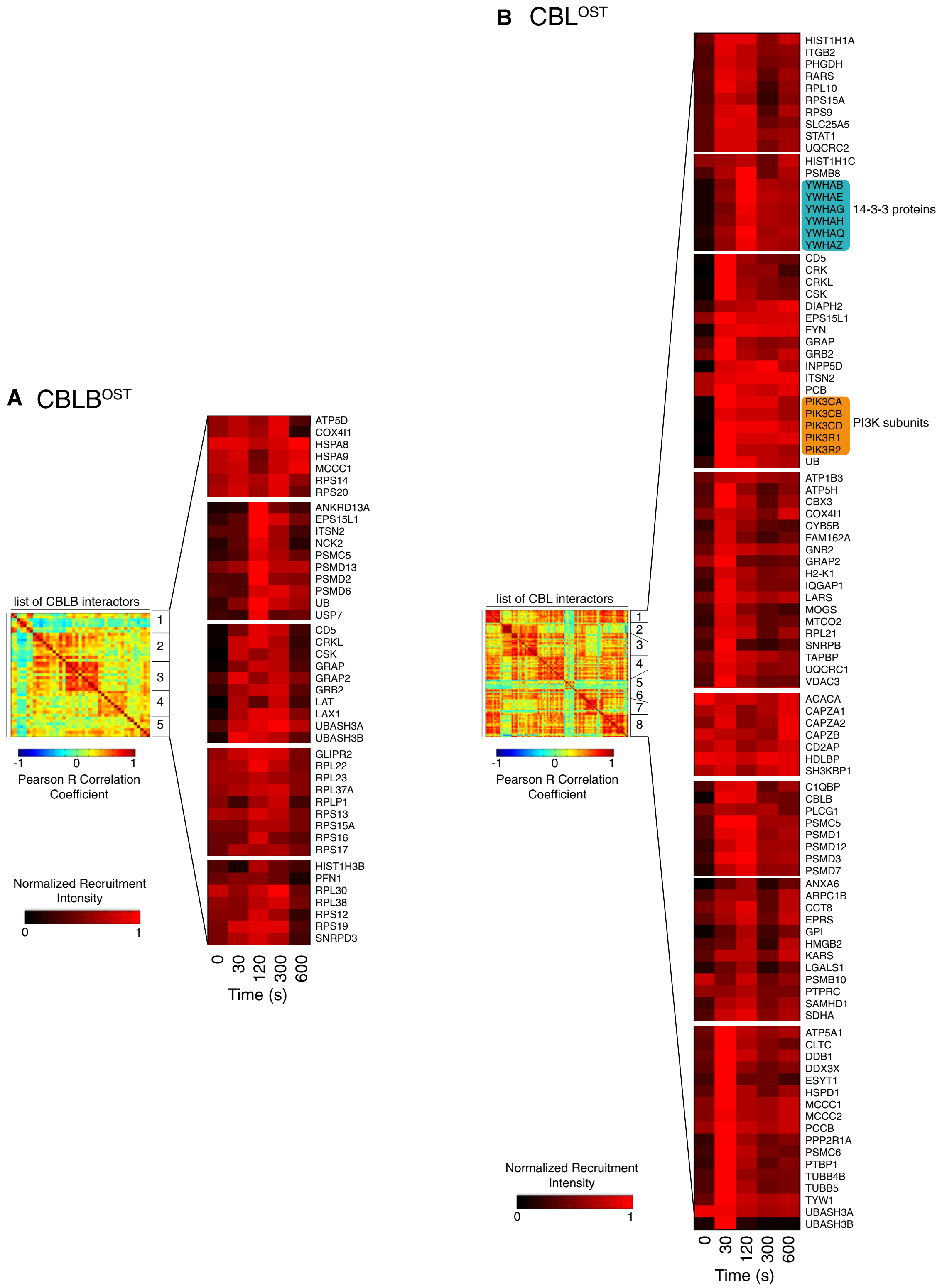

Figure EV4. K-means clustering analysis of the CBL and CBLB signalosomes.

A, B Representation of the CBLB (A) and CBL (B) correlation matrix (R_{ij}) partitioned into different clusters using a K-means clustering algorithm. The normalized recruitment intensity to the bait as a function of time is represented for the different interactors grouped into corresponding clusters. Within each cluster, interactors are listed in alphabetical order. Proteins from the 14-3-3 family and PI3K subunits are highlighted within the CBL signalosome.

© 2016 The Authors

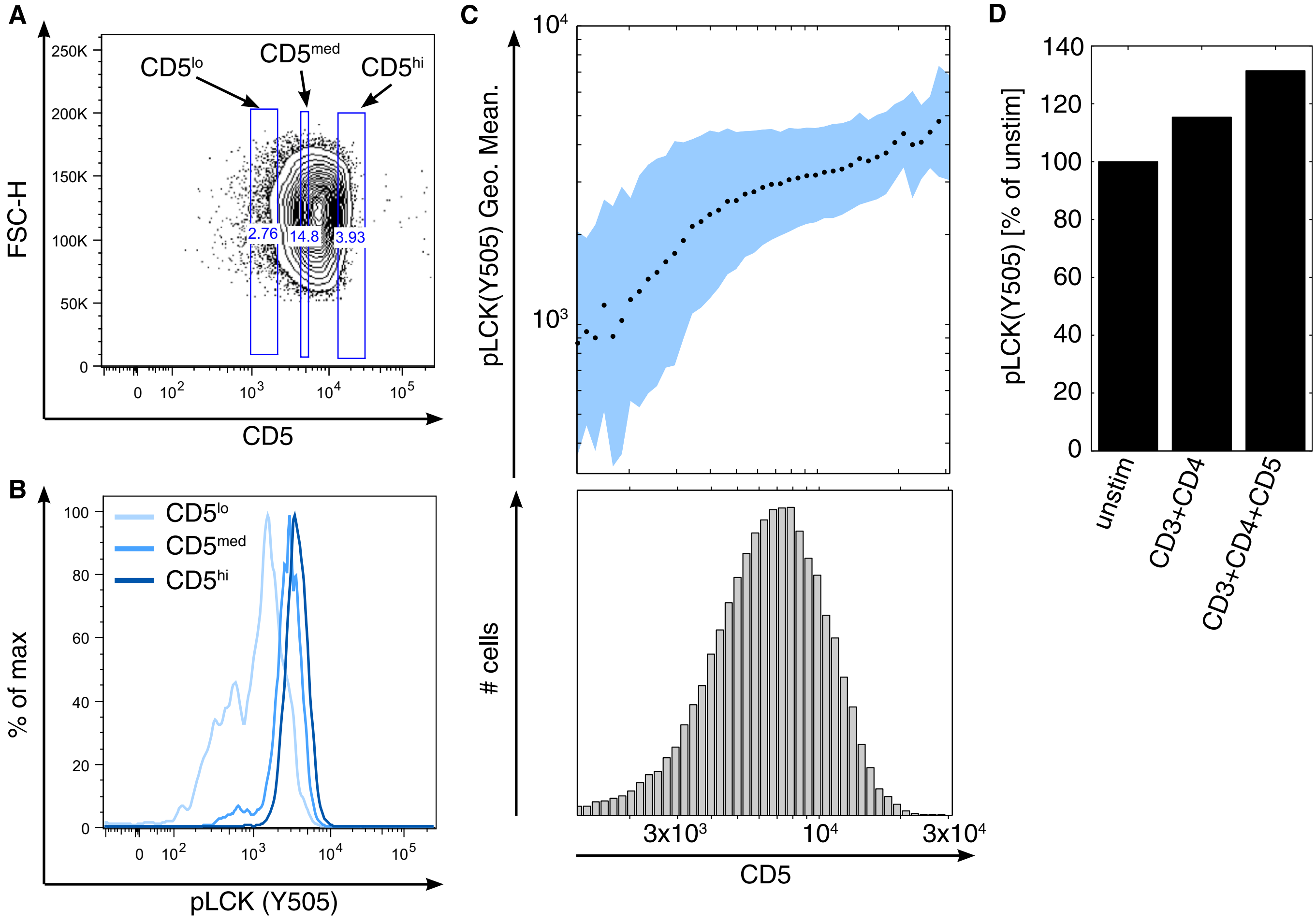

Figure EV5. CD5 contributes to control the phosphorylation of the negative-regulatory tyrosine found at position 505 of LCK via CSK.

A Expression of CD5 on short-term expanded $CD4^+$ T cells stimulated by cross-linking biotinylated anti-CD3 and anti-CD4 antibodies with streptavidin for 1 min at 37°C. The depicted gates define three populations of T cells with different expression of CD5 at their surface ($CD5^{lo}$, $CD5^{med}$ and $CD5^{hi}$). Numbers adjacent to outlined areas indicate percentage of cells.

B In addition to having been stained with anti-CD5, the cells described in (A) were permeabilized and stained with an anti-pLCK(Y505). The histogram represents the levels of phospho-LCK(Y505) found in the three populations defined in (A) on the basis of CD5 levels.

C The upper panel represents the mean (± SD) of the log fluorescence intensity of phospho-LCK(Y505) as a function of the geometric mean fluorescence intensity of CD5. Mean and SD were computed from populations of cells defined using a regular binning of the CD5 expression histogram (lower panel).

D Effect of CD5 cross-linking on the phosphorylation of Y505 of LCK. Short-term expanded $CD4^+$ T cells were stimulated with 2 µg biotinylated anti-CD3 plus 2 µg biotinylated anti-CD4 (as in A) in the presence or absence of 2 µg biotinylated anti-CD5 (clone 53-7.3). The intensity of phospho-LCK(Y505) is represented as percent of phospho-LCK(Y505) intensity in the unstimulated condition.

 © 2016 The Authors
